# Shared and distinct brain activation patterns of acupoints HT7, ST36, and KI4: a task-based fMRI study

**DOI:** 10.3389/fneur.2025.1596306

**Published:** 2025-06-26

**Authors:** Liyu Hu, Jinhuan Zhang, Xiaoxiong Wu, Xingxian Huang, Xinbei Li, Xingchen Liu, Hanqing Lyu, Nan Yang, Jinping Xu, Haibo Yu

**Affiliations:** ^1^Department of Acupuncture and Moxibustion, Shenzhen Traditional Chinese Medicine Hospital/The Fourth Clinical Medical College, Guangzhou University of Chinese Medicine, Shenzhen, China; ^2^Institute of Biomedical and Health Engineering, Shenzhen Institutes of Advanced Technology, Chinese Academy of Sciences, Shenzhen, China; ^3^Department of Rehabilitation, Shenzhen Bao'an Traditional Chinese Medicine Hospital, Shenzhen, China; ^4^Department of Radiology, Shenzhen Traditional Chinese Medicine Hospital/The Fourth Clinical Medical College, Guangzhou University of Chinese Medicine, Shenzhen, China; ^5^Hospital of Traditional Chinese Medicine of Zhongshan, Zhongshan, China

**Keywords:** task functional magnetic resonance imaging, acupuncture, acupoint specificity, brain network, deqi

## Abstract

**Objective:**

We aimed to investigate the shared and distinct of brain responses to manual acupuncture at HT7, ST36, and KI4.

**Methods:**

Sixteen healthy participants receiving acupuncture at HT7, 19 at ST36, and 16 at KI4. All subjects experienced deqi sensation without severe pain during the acupuncture sessions. Task-functional magnetic resonance imaging was performed to explore the common and specific brain activation networks. The activation networks were further decoded for functional characterization and analyzed for network components.

**Results:**

We found convergence deactivation in the left cerebellar crus II across all three acupoints. Additionally, specific patterns of deactivated brain network were observed: the left superior occipital gyrus, middle temporal gyrus, and inferior parietal lobule was predominately deactivated for the HT7, the left cerebellar Crus I and right middle occipital gyrus was primarily deactivated for the ST36, and the posterior cerebellum was mainly deactivated for the KI4. They were all involved in the default, attentional, and visual networks. In addition, the control network was also related to HT7, the limbic network to ST36, and the salient network to KI4. These functional networks were linked to spatial vision and cognition, with HT7 and ST36 also influencing emotional processing.

**Conclusions:**

All three acupoints caused shared deactivation in the left cerebellar Crus II, highlighting it as a significant region of neural convergence in acupuncture's brain modulation. HT7 and ST36 jointly influenced emotional processing, while KI4 targeted pain-cognition pathways. These findings support “acupoint-effect specificity” and guide clinical applications.

## 1 Introduction

According to traditional Chinese medicine (TCM) theory, acupoints play a crucial role in treating various clinical conditions. An increasing number of studies have been performed to explore the complex mechanisms underlying the efficacy of acupuncture, highlighting the significance of stimulating acupoints (1). However, the specificity of acupoints has long been controversial (2). Some studies suggested that the specificity of acupoints may not exist (3, 4) however, more neuroimaging studies have increasingly substantiated the specificity of acupoints (5). For example, the Shenmen (HT7) stimulation showed significantly different activated brain regions as compared to other acupoints associated with heart (6). Furthermore, studies on the Zusanli (ST36) revealed enhanced connectivity in limbic regions and reduced connectivity in sensory and frontal cortex areas as compared to non-acupoint stimulation, further emphasizing that acupuncture affects distinct brain networks in complex ways (7–9).

Most research mainly focused on single acupoint; however, certain points are often combined in clinical practice, leading to the need for understanding both the common and specific effects of each acupoint. For instance, research in hypertensive patients has highlighted distinct regional brain activity patterns when stimulating Taichong (LR3) and Taixi (KI3) (10). Similarly, in studies on pain-related disorders, acupoints such as Hegu (LI4), LR3, and ST36 have shown similar activation in the default mode network (DMN) while affecting specific brain regions uniquely (11, 12). Notably, acupuncture is widely used in the treatment of degenerative neurological conditions (13–15) and psychiatric conditions, such as depression and autism spectrum disorder (16). Among various acupoints used in these cases, HT7, ST36, and Dazhong (KI4) are particularly common (17, 18).

A meta-analysis showed ST36 could activate the bilateral left cerebellum, the bilateral Rolandic operculum, and the right supramarginal gyrus (19). In healthy individuals, stimulation of HT7 has been shown to activate the bilateral postcentral gyrus, inferior parietal lobule, inferior frontal gyrus, claustrum, insula, and cerebellum (20, 21). Additionally, HT7 acupuncture affects areas such as the medial prefrontal cortex, premotor cortex, amygdala, hippocampus, and thalamus, all of which collectively contribute to a reduction in smoking cravings (22). In individuals experiencing acute sleep deprivation, HT7 stimulation has been shown to broadly reverse abnormal functional connectivity disruptions across brain networks (23). Similarly, KI4 stimulation leads to activation in the right inferior frontal gyrus, right insular lobe, right thalamus, right middle frontal gyrus, and right orbitofrontal cortex (24). Based on the aforementioned findings, the acupoints ST36, HT7, and KI4 demonstrate activation in brain regions linked to sensory integration, emotional regulation, and executive function. In addition, activation of limbic structures such as the amygdala and hippocampus, particularly with HT7, suggests their involvement in emotional and cognitive processing. These overlapping neural pathways may explain why these acupoints often work together to treat neuropsychiatric disorders. However, research on the effects of HT7, ST36, and KI4 remains limited, with inconsistencies in experimental designs and statistical power across studies. As a result, it is still uncertain whether there are shared or distinct effects among these three acupoints in terms of their therapeutic mechanisms.

Therefore, we used task-based fMRI to investigate the neural activation patterns associated with manual acupuncture at acupoints HT7, ST36, and KI4 in healthy participants. We hypothesize that both common and specific brain regions are activated, with the areas consistently engaged across all three acupoints being involved in cognitive and emotional processing.

## 2 Methods

### 2.1 Participants

This study included 27 healthy, right-handed Han participants (six males, 21 females), aged 51–73 years (mean age ± SD: 60.67 ± 7.88). Participants were recruited from the Shenzhen Hospital of Traditional Chinese Medicine, and the study protocol was approved by the Ethics Committee of Shenzhen Traditional Chinese Medicine Hospital (Approval No. K2021-012-01) in accordance with the Declaration of Helsinki; all participants provided written informed consent. Individuals with neurological, psychiatric, or medical conditions, a history of drug abuse, head trauma with loss of consciousness, premenopausal women, or a BMI < 18.5 or ≥24 or contraindications to high magnetic fields were excluded.

Participants were randomly assigned to one of three stimulation orders—ST36 followed by KI4, ST36 followed by HT7, or KI4 followed by HT7—using computer-generated assignments in R (v4.0.5). An independent research assistant prepared consecutively numbered, opaque sealed envelopes containing each participant's order, ensuring allocation concealment. Immediately before the first needling session, the envelope was opened to determine the acupoint sequence. Each participant received acupuncture at two of these three acupoints according to this assigned sequence. A total of 18 participants received acupuncture at HT7, 19 at ST36, and 17 at KI4 (Supplementary Figure S1).

### 2.2 Acupuncture procedure

**Figure 1:**
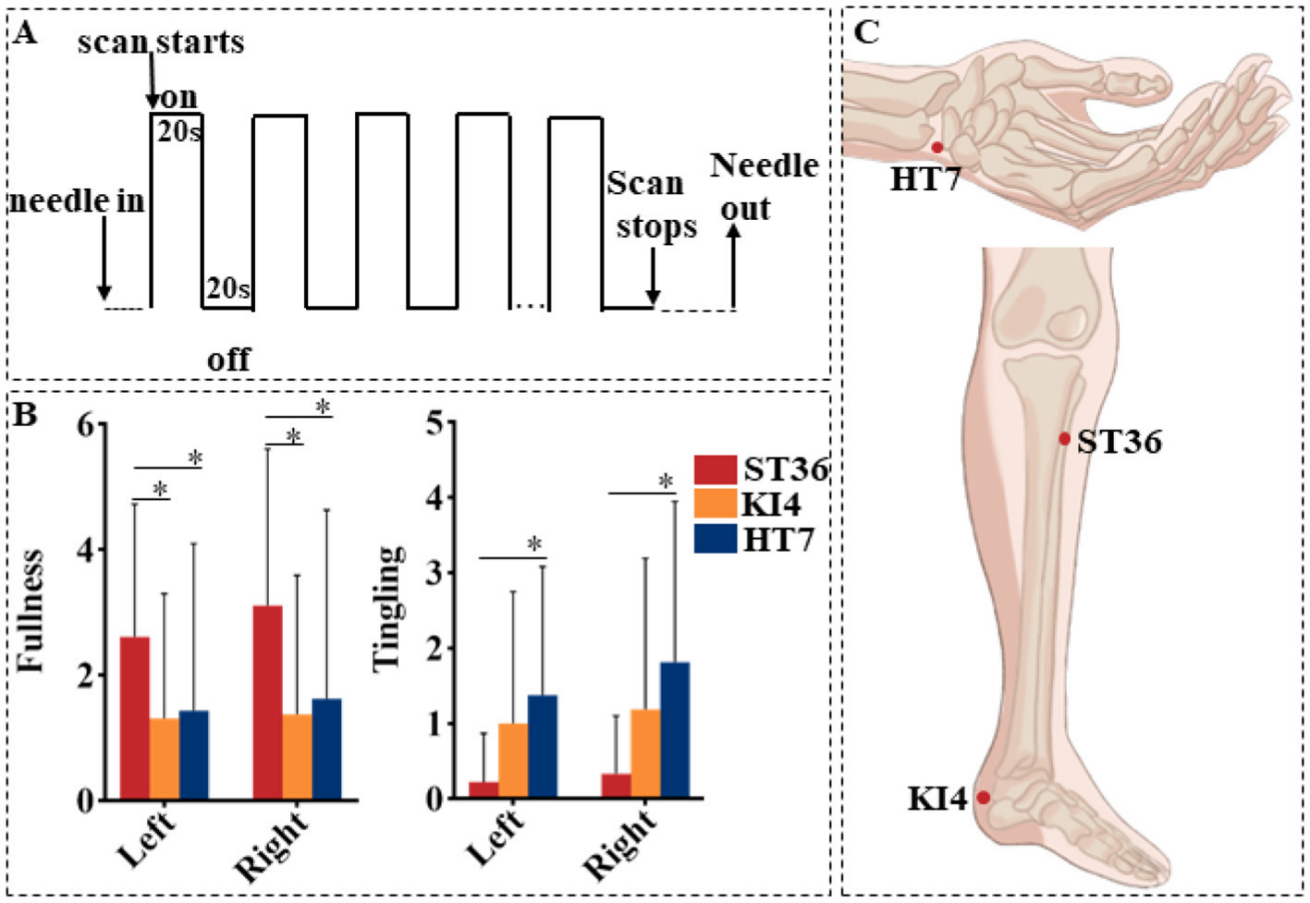
**(A)** The acupuncture stimulation paradigm. On denotes needle twirling and off denotes needle retention. **(B)** Differences of deqi scores among three groups. *Means *P* < 0.05. **(C)** The location of acupoints.

During a single session, acupuncture was administered bilaterally to a pair of acupoints selected from HT7, KI4, or ST36. The order of acupoint stimulation was determined by a randomized selection made prior to the session. Acupoints were located according to the guidelines from the National Standard of the People's Republic of China (GB/T 12346-2006; Figure 1C). Non-magnetic acupuncture needles (1/1.5 cun, No. 0Cr19Ni9N) from Suzhou Acupuncture Equipment Co., Ltd. (HuanQiu brand) were used for the procedure. The needle insertion depth ranged from 0.3 to 0.5 cun for HT7 and KI4, and 1 cun for ST36. Before the fMRI scan, the needles were inserted and rotated until the deqi sensation was elicited (25). During the 6-min scan, in the “on” phase, the acupuncturist rotated the needle at a frequency of 90–120 rotations per minute with a 90–180° for 20 s. This was followed by an “off” phase, during which the needle remained in place with no further manipulation for 20 s (Figure 1A). Each acupoint stimulation session involved one continuous scan of these nine “on-off” blocks, lasting a total of 360 s (6 min). To minimize after effects between two consecutive acupuncture sessions, participants rested in the scanner for 30 min between each session (26, 27). All acupuncture manipulations were performed by the same licensed acupuncturist (WXX).

### 2.3 Deqi measurements

After stimulation at each acupoint, participants were asked to report any sensations listed in the MGH Acupuncture Sensation Scale (MASS), including aching, soreness, deep pressure, heaviness, fullness or distension, tingling, numbness, dull pain, sharp pain, warmth, coolness, or any other specific sensation. If reported, participants rated each sensation on a scale from 1 to 10. A deqi response was defined as a total score of 3 or higher for any of the 12 sensations, excluding sharp pain (12). Only fMRI data from sessions where participants reported deqi were included in the analysis. To minimize confounding brain activations due to severe pain (28), any reported pain rating of 8 or higher during the scan was excluded from the analysis.

### 2.4 MRI data acquisition

All participants began the experiment following a 20-min rest period. Functional MRI (fMRI) images were acquired using a 3.0 Tesla MRI scanner (Siemens MAGNETOM Prisma). Participants lay supine with earplugs to reduce scanner noise, and their heads were stabilized with cushions. They were instructed to keep their eyes closed throughout each scan and remain awake and as still as possible. The fMRI scans used an echo-planar imaging sequence with the following parameters: repetition time (TR) = 2,000 ms, echo time (TE) = 30 ms, matrix size = 64 × 64, field of view = 240 × 240 mm, voxel size = 3.75 mm × 3.75 mm × 4.0 mm, flip angle = 90°, 37 axial slices with interleaved acquisition, slice thickness = 4 mm, no gap. T1-weighted structural images were also acquired with the following parameters: TR = 2,200 ms, TE = 2.45 ms, flip angle = 8°, acquisition matrix = 256 × 256, voxel size = 1 mm × 1 mm × 1 mm, 192 slices, and slice thickness = 1 mm. Acupuncture was performed at HT7, ST36, or KI4 during the fMRI scans, with each functional imaging session lasting 6 min.

### 2.5 Task-fMRI image analysis

Data pre-processing was conducted using RESTplus software (29). To mitigate the effects of magnetization non-equilibrium, the first 20 time points were excluded. Subsequently, each participant's 340 scans underwent a series of processing steps, including slice timing correction, realignment, co-registration with T1-weighted images, and segmentation. The processed images were then spatially normalized to the Montreal Neurological Institute standard echo-planar imaging template using DARTEL and resampled to a voxel size of 3 × 3 × 3 mm3. A Gaussian smoothing kernel with a full width at half maximum of 6 mm was applied to the images to enhance data quality. To mitigate the effects of head motion, data were included only from participants whose head motion did not exceed 3.0 mm in translation and 3.0° in rotation along each axis. As a result, one participant from the HT7 group was excluded due to excessive head movement.

Statistical analyses of task-fMRI were performed with Statistical Parametric Mapping software (SPM12, https://www.fil.ion.ucl.ac.uk/spm). The general linear model (GLM) was applied to the first-level analysis, with six motion parameters included as nuisance covariates. Our primary focus was on identifying brain regions activated by acupuncture. The contrast of interest was computed by subtracting baseline activity from the neural activation observed during needle manipulation. At the second level of analysis, we incorporated age and sex as covariates and conducted a one-sample *t*-test to evaluate activation patterns for each acupoint. Group differences were assessed using analysis of covariance (ANCOVA) and two-sample *t*-tests, as appropriate. Statistical significance was determined using Gaussian Random Field theory to correct for multiple comparisons, with a voxel-wise threshold of *P* < 0.001 and a cluster-level significance of *P* < 0.05, and a minimum cluster size of 90 voxels.

### 2.6 Commonality and specificity of activated brain networks

The investigation of commonalities in brain networks was conducted using conjunction analysis to identify regional overlaps in brain responses. Subsequently, by subtracting co-activation from the activation patterns associated with each acupoint, relatively specific activation patterns for these acupoints were identified (12).

### 2.7 Functional characterization of activated brain networks

We utilized the Meta-Analytic Connectivity Modeling (MACM) toolbox (30), specifically employing the Mango Behavioral Analysis v3.1 and Paradigm Analysis v1.6 plugins, to decode the functional profile of each identified cluster. For each behavioral subdomain or paradigm class, the probability of activation in our cluster—conditioned on that domain/paradigm—is contrasted against the baseline probability of cluster activation across all BrainMap experiments. All reported functional associations were further subjected to false discovery rate (FDR) correction at *P* < 0.05.

### 2.8 Decoding of activated brain network mappings

Thomas Yeo et al. (31) segmented the cerebral cortex into 17 distinct components based on functional connectivity to represent networks associated with different functions. Similarly, the cerebellum has been parcellated into 17 components corresponding (32). In our study, we used the above atlas to perform network component analysis on the brain regions activated by acupuncture. For each acupoint, we overlaid its activation mask onto the Yeo-17 network atlas. We computed the proportion of voxels falling within each of the 17 canonical networks. Networks with < 1% overlapping voxels were not reported.

### 2.9 Statistical analysis

Statistical analysis was conducted using SPSS version 26.0. First, we performed a normality test using the Shapiro–Wilk test, which showed that only age followed a normal distribution. Consequently, comparisons of age between groups were performed utilizing analysis of variance (ANOVA). For variables such as years of education, Mini-Mental State Examination (MMSE) scores, Montreal Cognitive Assessment (MoCA) scores, and MASS scores, the Kruskal–Wallis *H* test was employed. The distribution of sex was examined using the chi-square test (χ^2^).

## 3 Results

### 3.1 Demographics and deqi measurements

Two participants were excluded from The HT7 group due to excessive head movement and severe pain, whereas one participant was excluded from the KI4 group for excessive head movement. The final analysis included 16 participants in both the HT7 and KI4 groups and 19 in the ST36 group. No significant difference was showed in terms of age, sex, years of education, cognitive function assessments, and MASS scores among the three groups (Table 1).

**Table 1:** Characteristics of three groups.

**Item**	**Zusanli**	**Dazhong**	**Shenmen**	***P* value**
Sex (male/female)	4/15	4/12	4/12	0.950^a^
Age	60.05 ± 7.90	59.19 ± 7.91	60.59 ± 6.73	0.866^b^
Education	12.11 ± 2.73	13.13 ± 3.01	12.53 ± 3.34	0.614^c^
MMSE	28.95 ± 1.03	28.56 ± 1.26	28.53 ± 1.13	0.498^c^
MoCA	27.37 ± 0.96	27.63 ± 0.81	27.35 ± 1.06	0.571^c^
MASS_L	6.68 ± 5.88	5.88 ± 4.69	6.53 ± 5.34	0.944^c^
MASS_R	7.53 ± 50.63	6.44 ± 5.57	7.82 ± 5.62	0.608^c^

^a^Denotes the chi-square test; ^b^represents the one-way ANOVA test; ^c^ represents the Kruskal-Wallis H test; MASS_L is the total MASS score for the left body; MASS_R is the total MASS score for the right body.

The sensation of fullness was significantly higher at bilateral ST36 as compared to KI4 and HT7. The sensation of tingling was higher at bilateral HT7 than at ST36 (Figure 1B).

### 3.2 Deactivated brain networks

**Figure 2:**
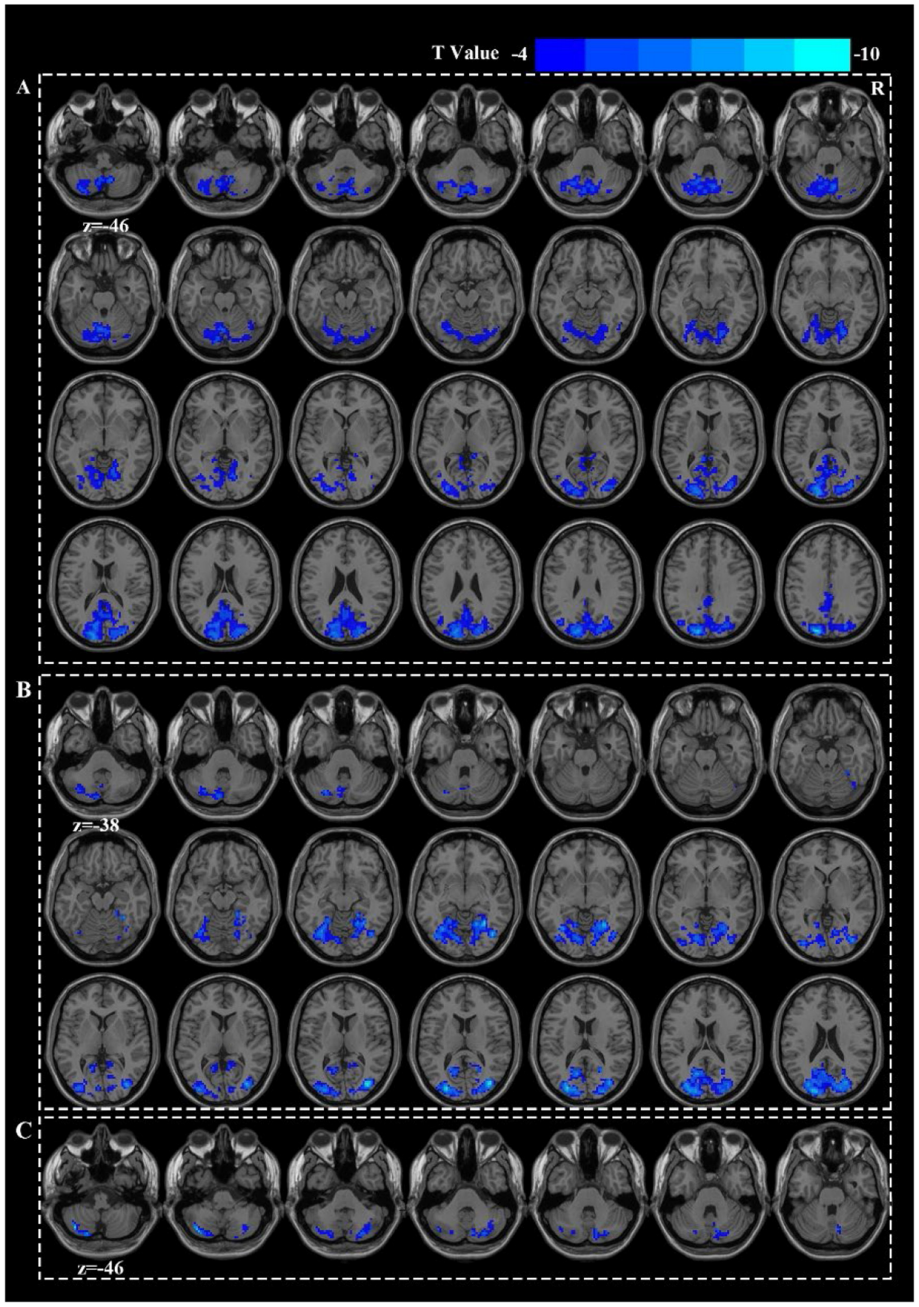
Active patterns for HT7 **(A)**, ST36 **(B)**, and KI4 **(C)**.

Acupuncture at ST36 elicited deactivation in the right middle occipital gyrus (MOG.R) and the left cerebellar crus I (Figure 2B). Acupuncture at HT7 led to deactivations in the left superior occipital gyrus (SOG.L), left middle temporal gyrus (MTG.L), and left inferior parietal lobule (IPL.L) (Figure 2A). Acupuncture at KI4 were restricted to the deactivation of the left cerebellar crus II and the right cerebellar crus I (Figure 2C). However, there were no significant differences in the deactivated brain regions either among the three groups or between any two groups (Figure 1, Supplementary Table S1).

### 3.3 Commonly deactivated brain network

**Figure 3:**
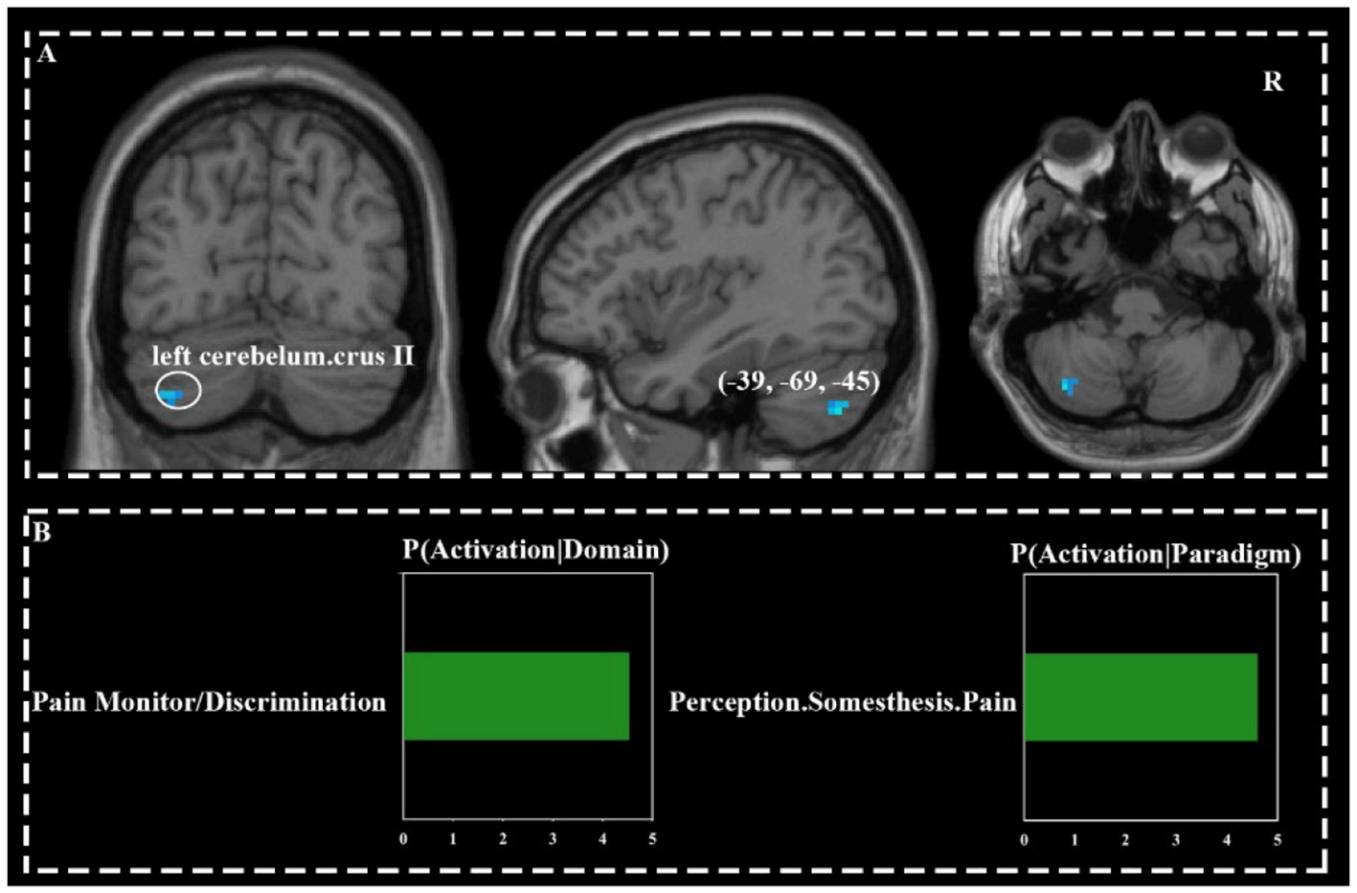
Commonly activated brain regions across three acupoints **(A)** and their functional characteristics **(B)**.

The left cerebellar crus II was co-deactivated across three acupoints (Figure 3A).

### 3.4 Specificity of the brain network

To rule out brain activation caused by pain during acupuncture, we compared the activation networks of each acupoint with pain-related brain regions (extracted from Neurosynth using the Pain Uniformity Test map, thresholded at a false discovery rate (FDR) of *q* < 0.01; see https://www.neurosynth.org/analyses/terms/pain). We identified overlapping regions where the cluster size exceeded 10 voxels. The results showed no overlap between pain-related brain areas and the regions deactivated by ST36 and KI4. However, HT7 showed overlapping deactivation in the right cerebellar lobule IX and the left cerebellar lobule VI with pain-related areas (Supplementary Table S2).

The specific activation network for each acupoint was defined by subtracting the overlapping voxels of the pain network and the co-activation network from each acupoint's activated network. The specific deactivation network for HT7 primarily included the bilateral middle occipital gyrus, bilateral superior occipital gyrus, bilateral precuneus, bilateral cuneus, bilateral lingual gyrus, bilateral posterior lobe of the cerebellum, the left middle temporal gyrus, and the left inferior parietal lobule. For ST36, the specific network mainly involved the left cerebellum crus I, bilateral middle occipital gyrus, bilateral superior occipital gyrus, bilateral cuneus, bilateral lingual gyrus, and bilateral fusiform gyrus. The specific deactivation network for KI4 was primarily located in the posterior lobe of the cerebellum, mainly including the bilateral cerebellum crus II and the right cerebellum crus I (Supplementary Figure S2).

### 3.5 Functional characterization of deactivated brain networks

Behavioral domain analysis indicated that the common deactivation network was most associated with pain monitoring/discrimination (*Z* = 4.61). The deactivation network for HT7 was associated with six subdomains, including two subdomains related to vision under perception, three cognition subdomains, and one interoception subdomain. The strongest association was found with the interoception subdomain of hunger (*Z* = 2.06), and the cognitive domains were broadly related to language and spatial processing. The deactivation network for ST36 involved four domains: interoception, emotion, cognition, and perception, spanning a total of seven subdomains. The strongest association was also with the interoception subdomain of hunger (*Z* = 2.15). The deactivation network for KI4 was focused on the cognitive subdomain of semantics, with an association strength of *Z* = 1.64.

**Figure 4:**
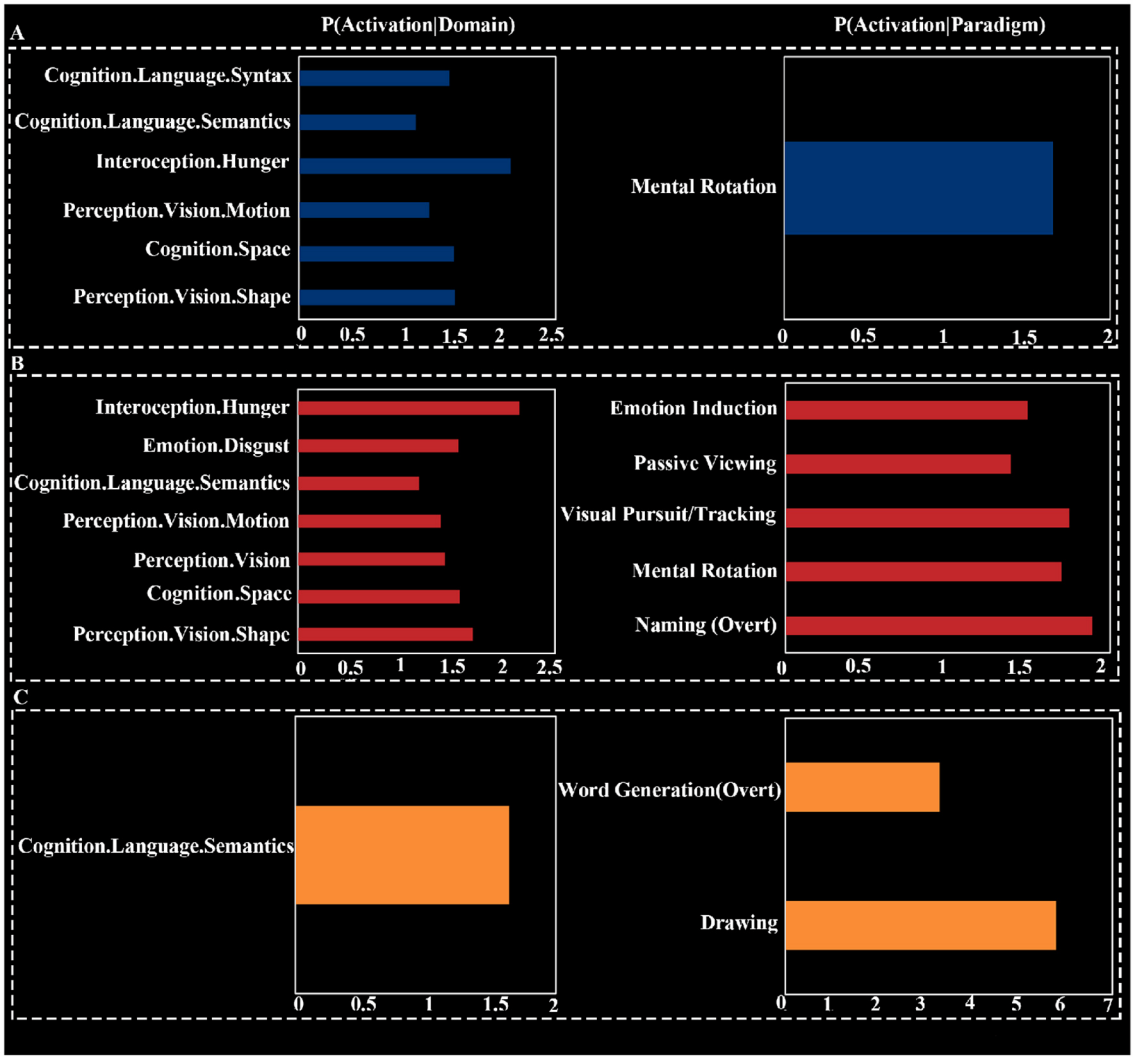
Functional characterization for HT7 **(A)**, ST36 **(B)**, and KI4 **(C)**.

Paradigm class analysis showed that the common deactivation network had the strongest association with the “Pain Monitor/Discrimination” paradigm class (*z* = 4.53; Figure 3B). The deactivation network for HT7 was primarily associated with the “Mental Rotation” paradigm class (*z* = 1.65). The deactivation network for ST36 was linked to five different paradigm classes, with the strongest association being with “Naming (Overt)” (*z* = 1.89). The deactivation network for KI4 was related to two paradigm classes: “Drawing” (*z* = 5.79) and “Word Generation (Overt)” (*z* = 3.30; Figure 4).

### 3.6 Results of deactivated brain network components

**Figure 5:**
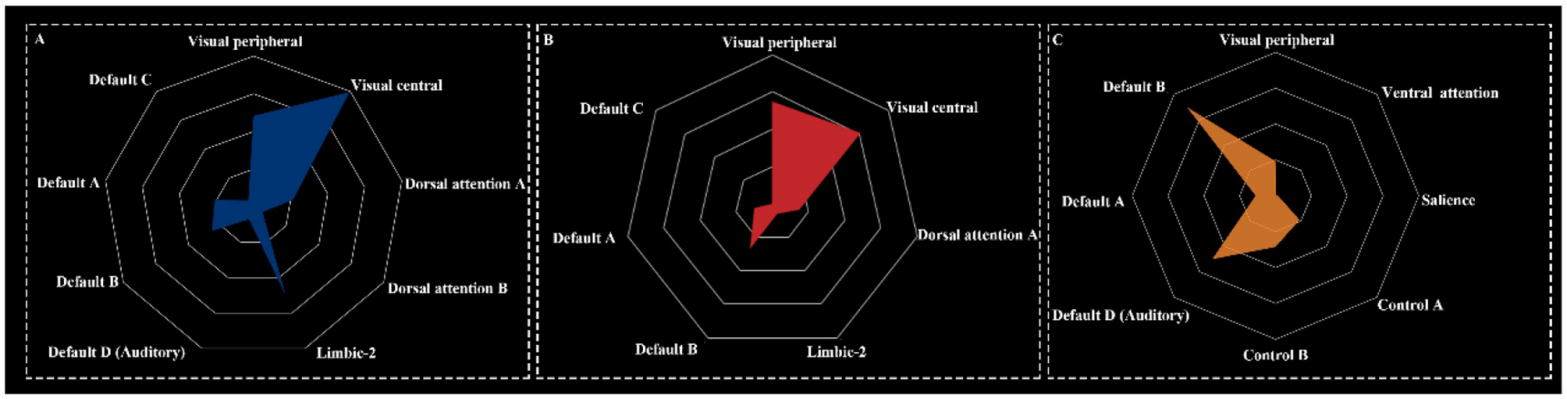
Network components for HT7 **(A)**, ST36 **(B)**, and KI4 **(C)**.

The deactivation networks for HT7 and ST36 exhibited similar components, encompassing the visual network, dorsal attention network (DAN), limbic network, and default mode network. In contrast, the deactivation network for KI4 was broader, incorporating the visual network, ventral attention network, salience network, control network, and default mode network (Figure 5).

## 4 Discussion

In this study, we explored the common and specific brain activation patterns following acupuncture at three acupoints commonly applied in treating neuropsychiatric conditions. With a strict statistical threshold, we observed significant and widespread brain deactivation across task-based MRI scans (33). Acupuncture at HT7 showed the broadest deactivation, followed by ST36, whereas the effects of KI4 were predominantly localized to the cerebellum, which may be related to the higher scores of deqi in HT7 and ST36. Notably, all three acupoints led to deactivation of the posterior cerebellum; however, HT7 and ST36 also commonly deactivated additional brain regions. Consistent with previous findings, our deactivated brain network mapping revealed that acupuncture suppresses the default mode network (34). Importantly, we also found that acupuncture of a single acupoint in HT7, ST36, and KI4 resulted in the concurrent deactivation of multiple brain networks (21, 24, 35). This deactivation pattern was consistent across different acupoints, with overlapping deactivation observed in the visual, attentional, and default mode networks. Functional characterization showed that these three acupoints are predominantly linked to processes involving cognition, perception, interoception, and emotion.

### 4.1 Co-deactivation of brain networks

Stimulation of HT7, ST36, and KI4 consistently deactivated the left cerebellar crus II (Figure 3A), a posterior cerebellum area, which has been reported of deactiviation in individuals with cognitive impairments (36) and psychiatric disorders (37, 38). Emerging research highlights non-motor functions of the cerebellum, emphasizing its roles in cognition and emotion (39, 40). A meta-analysis of roughly 148 fMRI studies showed cerebellar crus II as key to social mentalizing and emotional cognition (41). This region is also involved in cognitive tasks such as semantic fluency, category switching, and spatial skills (42).

Acupuncture at HT7 has been reported to deactivate posterior cerebellar regions in individuals with alcohol dependence (20). Other findings are consistent with prior evidence that cerebellar deactivation occurs following acupuncture at ST36, regardless of the presence of the deqi sensation (43). Notably, such activation has only been observed in healthy subjects (44), suggesting potential underlying differences in cerebellar responsivity across health states.

Functional characterization showed that the deactivated areas were primarily associated with pain perception (Figure 3B). This may be caused by the sensation of pain during acupuncture, and fMRI studies have shown that the cerebellum tends to be deactivated during pain (45–47).

### 4.2 Specificity characterized in the deactivated network of HT7

Acupuncture at HT7 caused the widest range of deactivation, involving brain areas such as the occipital lobe, precuneus, cuneus, lingual gyrus, posterior cerebellum, middle temporal gyrus, and inferior parietal lobule. Yujuan (48) research highlights that the occipital and frontal lobes play a central role in insomnia, with HT7 acupuncture shown to regulate blood oxygen metabolism within these areas. Results from Electroencephalography further supports this by showing increased synchronization in temporo-occipital regions within the theta and alpha frequency bands after HT7 stimulation (49). In animal studies (50), acupuncture at HT7 has been found to modulate cerebral glucose metabolism in the temporal lobe and enhance memory in Alzheimer's disease model rats (51). Our findings align with prior studies that showed deactivation in the left inferior parietal lobule following HT7 acupuncture (6). However, some other studies reported activation in this area, potentially due to differing subject conditions—young healthy individuals (21) in one case and alcohol-dependent individuals in another (20). This suggests that HT7 may produce variable effects depending on the physiological or pathological state of the individual.

In the network component analysis, we observed that HT7 stimulation primarily deactivated regions associated with the visual network, the dorsal attention network (DAN), the default mode network (DMN), and the limbic network. Among them, the superior occipital gyrus is a core region for visual information processing in the central visual network (52). The middle temporal gyrus and the inferior parietal lobe are also involved and formed part of the peripheral visual network responsible for higher-level visual processing. These regions contribute to conscious and unconscious shifts in attention triggered by gaze (53). Vision has been strongly linked to psychiatric disorders such as depression and eating disorders (54, 55). In depressed patients, the middle occipital gyrus shows abnormal activation, possibly reflecting the role of the temporo-occipital cortex in transmitting external stimuli to emotionally relevant regions (56). The middle temporal gyrus and inferior parietal lobule also synergize in semantic processing (57), and in addition, the temporal lobe and limbic network are commonly affected simultaneously in patients with mild cognitive impairment (58).

The inferior parietal lobule is a key component of the default and dorsal attention networks (59), playing a central role in top-down attention and spatial orienting (50). Given the important functions of the default mode network in conscious awareness, emotion processing, attentional control, and working memory, alterations in its connectivity are frequently observed in psychiatric disorders (60). Functional mapping of HT7-activated brain regions further confirmed the involvement of these networks in domains including visual shape recognition, spatial and motion perception, semantic processing, and interoceptive hunger. Among these, interoceptive awareness has been associated with various psychiatric and neurodegenerative disorders, possibly due to its effects on cognition, decision-making, and memory (58).

Our paradigm analysis of HT7 deactivation regions suggests that needling HT7 affects spatial cognition and visual processing abilities, which typically decline with age. This decline in tasks involving mental rotation and visualization may partially explain the benefits of acupuncture at HT7 on visual spatial and cognitive abilities in older subjects.

### 4.3 Specificity characterized in the deactivated network of ST36

Acupuncture at ST36 caused deactivation of brain regions including posterior cerebellum, middle occipital gyrus, superior occipital gyrus, cuneus, lingual gyrus, and fusiform gyrus. Meta-analyses have shown that acupuncture at ST36 typically activates the bilateral cerebellum, which contrasts with our findings (19, 35). This discrepancy likely arises from key methodological differences. Meta-analyses reporting cerebellar activation predominantly featured younger participants; Zhang et al. also included studies employing electroacupuncture. In contrast, our study focused on an older cohort (51–73 years) and utilized exclusively manual acupuncture with repeated stimulation (nine times) over a 6-min fMRI scan. This approach differs from some studies in Huang et al. (35), which involved younger participants, some single-instance stimulations, and shorter fMRI scan durations (< 5 min). These significant variations in participant demographics (age), acupuncture techniques (manual vs. electroacupuncture), and fMRI experimental design (stimulation frequency, scan duration) are crucial factors likely contributing to the differing cerebellar activity patterns observed at ST36 and warrant careful consideration when comparing study outcomes. Furthermore, subgroup analyses indicate that changes in cerebellar signaling might be more variable, while a decrease in signal in the middle occipital gyrus emerged as a reliable finding (61). Our results, however, align with those of a study that employed a similar design. This study reported significant deactivation effects in the cerebrum, and brainstem following acupuncture at ST36, particularly in healthy individuals who experienced deqi without severe pain (62). Bai et al. (63) proposed that acupuncture may produce delayed effects, leading to an increase in baseline levels and a subsequent deactivation. It is important to carefully explain that acupuncture at ST36 could modulate the function of the left cerebellar crus I. However, specific research paradigms must be taken into account when interpreting the exact nature of these activation patterns.

A consistent observation across studies, including ours, is the decreased nodal degree in the occipital lobe following acupuncture (64). Both classical general linear model (GLM) and independent component analysis (ICA) methods revealed significant deactivation in the occipital cortex (65), particularly with lifting-twisting manipulation (66). This deactivation is likely linked to a reduction in visual cortex activity when cognitive load—particularly related to attention and executive function—increases (67). Such findings may reflect the dynamic interplay between sensory processing and higher-order cognitive demands during acupuncture.

In addition to modulating the visual network, acupuncture at ST36 also exerted an influence on the limbic network, the DMN, and the DAN, corroborating previous meta-analytic findings (35). Significantly, acupuncture at ST36 resulted in a more pronounced deactivation of the DMN compared to other acupoints (68). Prior research has demonstrated that acupuncture can induce deactivation of the limbic-paralimbic-neocortical network (LPNN), with tactile stimulation eliciting less deactivation of the limbic network than needling (11, 69). In terms of functional domains, acupuncture at ST36, although deactivating fewer voxels compared to HT7, was engaged a broader array of functional domains. Beyond its effects on visual, spatial, and motion perception, as well as semantic processing, acupuncture at ST36 significantly deactivated regions associated with interoception, particularly the sensation of hunger. This observation aligns with TCM, which posits that ST36 is integral to the regulation of spleen and stomach functions (35), potentially accounting for hunger-related activation. Furthermore, the regulation of emotion, as observed within the functional domain, may be associated with acupuncture's influence on the limbic-paralimbic-neocortical network, possibly through the deactivation of regions involved in emotional processing (69).

The overlap of voxels is prevalent in the deactivation regions of HT7 and ST36. To further investigate the commonalities between these two acupoints, we identified their involvement in cognition, internal sensation, and visuospatial processing (Supplementary Figure S3). This may elucidate why these acupoints are frequently paired and utilized together. However, some researchers have suggested that the specificity of acupoints is reflected not only in the area of regulation but also in the degree of regulation (68). This aspect warrants further investigation in future studies. Moreover, MACM inherently relies on the meta-analytic aggregation of published tasks and behavioral annotations, which may vary in quality and scope. Such reverse-inference approaches are particularly sensitive to threshold selection and database coverage biases. Therefore, while our MACM results suggest potential links to hunger-related interoception at HT7 and ST36, and to semantic processing at KI4, future work is needed to validate these associations with independent task-based or connectivity-based paradigms. Consequently, these results should be viewed as preliminary and hypothesis-generating rather than conclusive.

### 4.4 Specificity characterized in the deactivated network of KI4

Acupuncture at KI4 primarily led to deactivation in the posterior cerebellum, with significant effects observed in the left second cerebellar ridge and the right first cerebellar ridge. While task-state fMRI studies on KI4 are limited, TCM theory suggests that acupoints exert two types of effects: those related to the meridian the point belongs to, and those linked to the specific location of the acupoint. Therefore, KI4 shares functional similarities with KI3, another acupoint on the same meridian. Consistent with our findings, acupuncture at KI3 also resulted in deactivation of the posterior cerebellum, inhibiting regions involved in emotional processing, attention, and phonological and semantic processing (70). Studies have shown that damage to the posterior cerebellum increases the risk of autism (71), and KI4 is commonly used in clinical practice for treating autism (17). Notably, effective connections between cerebellar Crus I and regions involved in visual attention, such as the visual cortex and posterior parietal lobes, have been observed (72). The cerebellum is also significantly deactivated during auditory attention tasks (73). Through its connections with the cerebral cortex, the cerebellum plays a critical role in cognitive functions, including attention, executive control, language, and working memory (74, 75). In typically developing individuals, cerebellar Crus I/II are functionally connected to both the default mode network and the fronto-parietal network, with substantial overlap in areas involved in language processing (74). These findings further support results from the network component analysis for KI4. Additionally, viral tract-tracing studies have identified anatomical connections between right Crus I and Brodmann area 46, as well as other language-related regions of the cerebral cortex (76). Both Crus I and II are activated during language processing tasks, with contralateral connections between the cerebellum and cerebral cortex, reflecting right-lateralized cerebellar involvement in language processing that mirrors the left-lateralized processing in the cortex. Damage to the right posterior cerebellum can result in deficits in both receptive and expressive language (77), which may explain the functional characteristics of brain regions activated during language-related tasks. In functional characterization analyses, activated areas were also associated with visuospatial processing, likely due to crus I/II's interconnections with frontal and parietal association areas, including regions important for processing visual body motion, such as the superior temporal sulcus (78).

While the deactivation regions of KI4 were restricted to the cerebellum, the functional extension of the cerebro-cerebellar circuit enables the cerebellum to modulate broad networks, thereby contributing to language processing, particularly in the domain of semantics.

## 5 Limitations

There are some limitations in our study. First, the sample size was relatively small, and skewed toward female participants; however, we included sex as a covariate in our statistical analyses to mitigate this imbalance. We plan to expand the sample size in future studies to enhance the generalizability of our findings. Second, we used a classic block design, there is a possibility of a delayed response to the stimulus effect, which warrants further investigation to ascertain its occurrence. Third, the effects of acupuncture may vary between healthy individuals and patients. Therefore, the results should be interpreted with caution when applied to patients. Future studies will include both healthy individuals and patients to explore these potential differences. Fourth, this study lacked a sham acupuncture or non-acupoint control group. Consequently, we cannot definitively attribute the observed brain responses solely to acupoint-specific effects, as distinct from general somatosensory stimulation or placebo effects. Future studies will incorporate appropriate control conditions to address this. Fifth, we did not include specific behavioral or psychometric scales to directly validate the functional interpretations derived from fMRI activation patterns. Future research should incorporate such assessments to strengthen the link between observed neural activity and functional outcomes.

## 6 Conclusion

This study employed task-functional MRI to reveal shared and distinct brain activation patterns induced by acupuncture at HT7, ST36, and KI4. All three acupoints elicited convergent deactivation in the left cerebellar Crus II, indicating its potential role as a neural hub for acupuncture-mediated brain modulation. Unique network profiles were identified: HT7 linked to deactivation in the left superior occipital gyrus, middle temporal gyrus, and inferior parietal lobule; ST36 specifically involved the left cerebellar Crus I and right middle occipital gyrus; KI4 predominantly affected the posterior cerebellum. Functional decoding highlighted the default, attentional, and visual networks as common regulators across acupoints, with HT7 additionally engaging the control network (spatial vision/emotion), ST36 the limbic network (visceral/emotional integration), and KI4 the salience network (pain/cognition).

These findings systematically demonstrate that acupoints achieve multi-target effects through distinct neural mechanisms: HT7 and ST36 synergistically modulate emotional processing, while KI4 prioritizes pain-cognition pathways. These findings offer preliminary neuroscientific insights suggestive of the acupoint-effect specificity principle. Future studies should expand sample sizes, include clinical populations, aim to directly correlate such neuroimaging findings with relevant clinical, behavioral, or psychometric outcomes to validate these potential therapeutic connections and genuinely inform clinical precision.

## Data Availability

The original contributions presented in the study are included in the article/Supplementary material, further inquiries can be directed to the corresponding authors.
