# Supplementary material for: Shared and distinct brain activation patterns of acupoints HT7, ST36, and KI4: a task-based fMRI study
Source: Front Neurol. 2025 Jun 26;16:1596306. doi: 10.3389/fneur.2025.1596306 (PMC12241811; doi:10.3389/fneur.2025.1596306)
Supplement: Supplementary file 1 [file Table_1.docx]

sTable 1 Activation Brain Regions Identified in the Three Groups.

| Acupoints | Brain region  (Abbreviation) | MNI coordinates  (x,y,z) | Voxel size | T |
| --- | --- | --- | --- | --- |
| HT7 | SOG.L | (-21, -81, 39) | 4362 | -11.0835 |
|  | MTG.L | (-57, -21,-6) | 95 | -4.7373 |
|  | IPL.L | (-54, -48,3) | 93 | -5.6566 |
| ST36 | CC1.L | (-27, -75, -33) | 137 | -5.2213 |
|  | MOG.R | (36, -78, 9) | 2698 | -7.6878 |
| KI4 | CC2.L | (-39, -69, -45) | 109 | -7.4857 |
|  | CC1.R | (27, -78, -36) | 163 | -5.5132 |

sTable2 Overlap Between HT7 Activation Regions and Pain Network.

| Brain region | Abbreviation | MNI coordinates  (x,y,z) | Voxel size |
| --- | --- | --- | --- |
| Right Cerebellum Lobule XI | Cere9.R | (9,-60,-45) | 13 |
| Left Cerebellum Lobule VI | Cere6.L | (-27,-69,-24) | 17 |


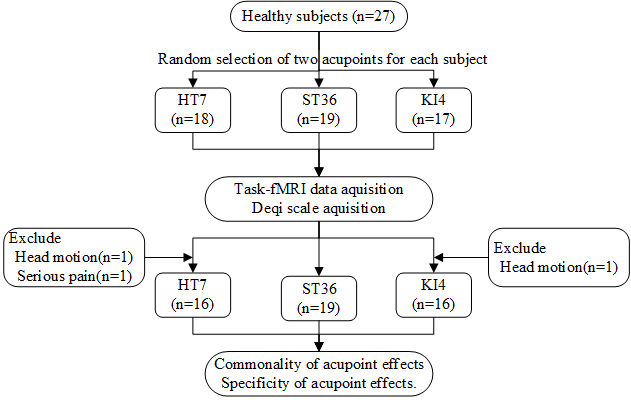


sFigure1 Flow diagram


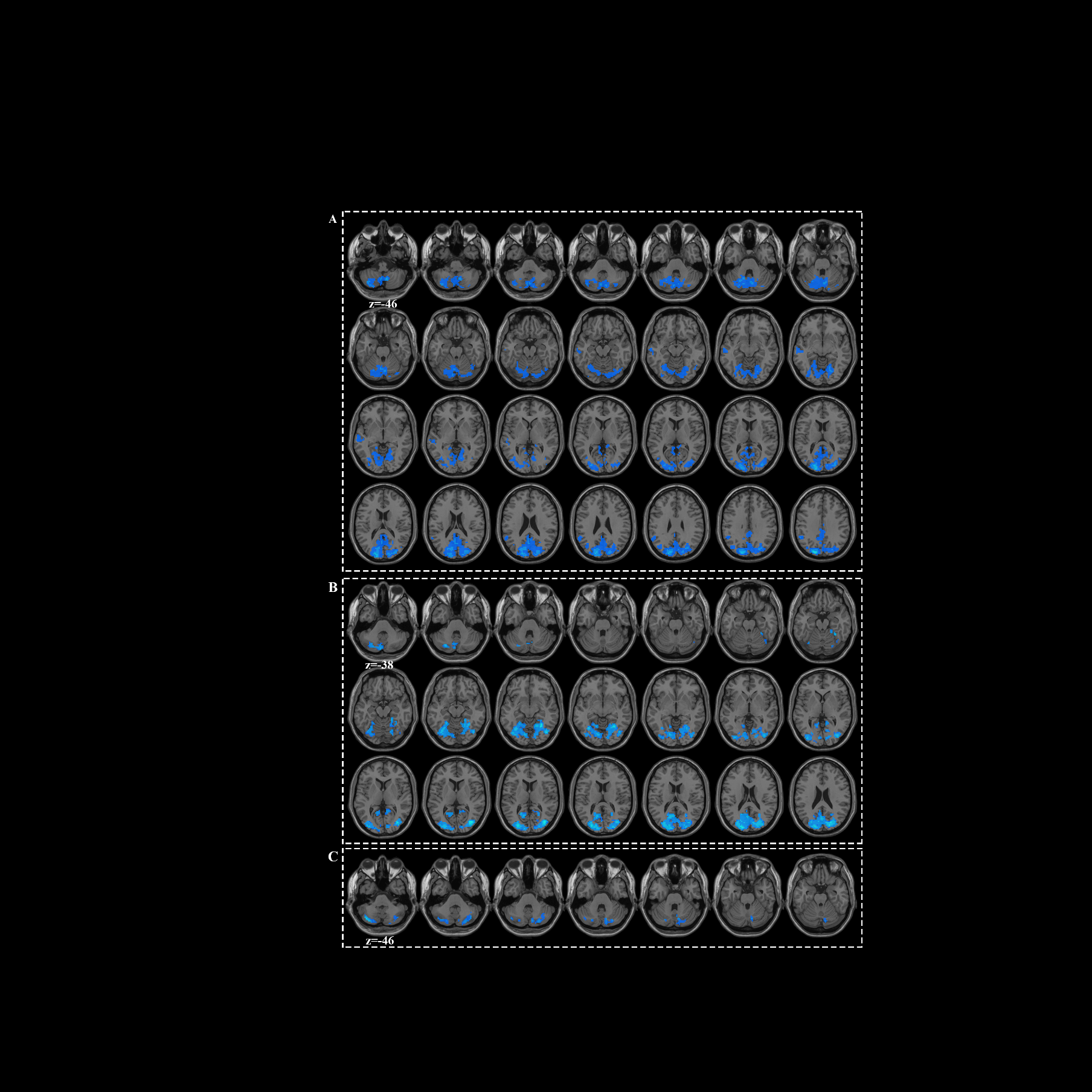


sFigure 2. Acupoint-Specific Activation Patterns. A) HT7-Specific Activation Pattern B) ST36-Specific Activation Pattern C) KI4-Specific Activation Pattern.


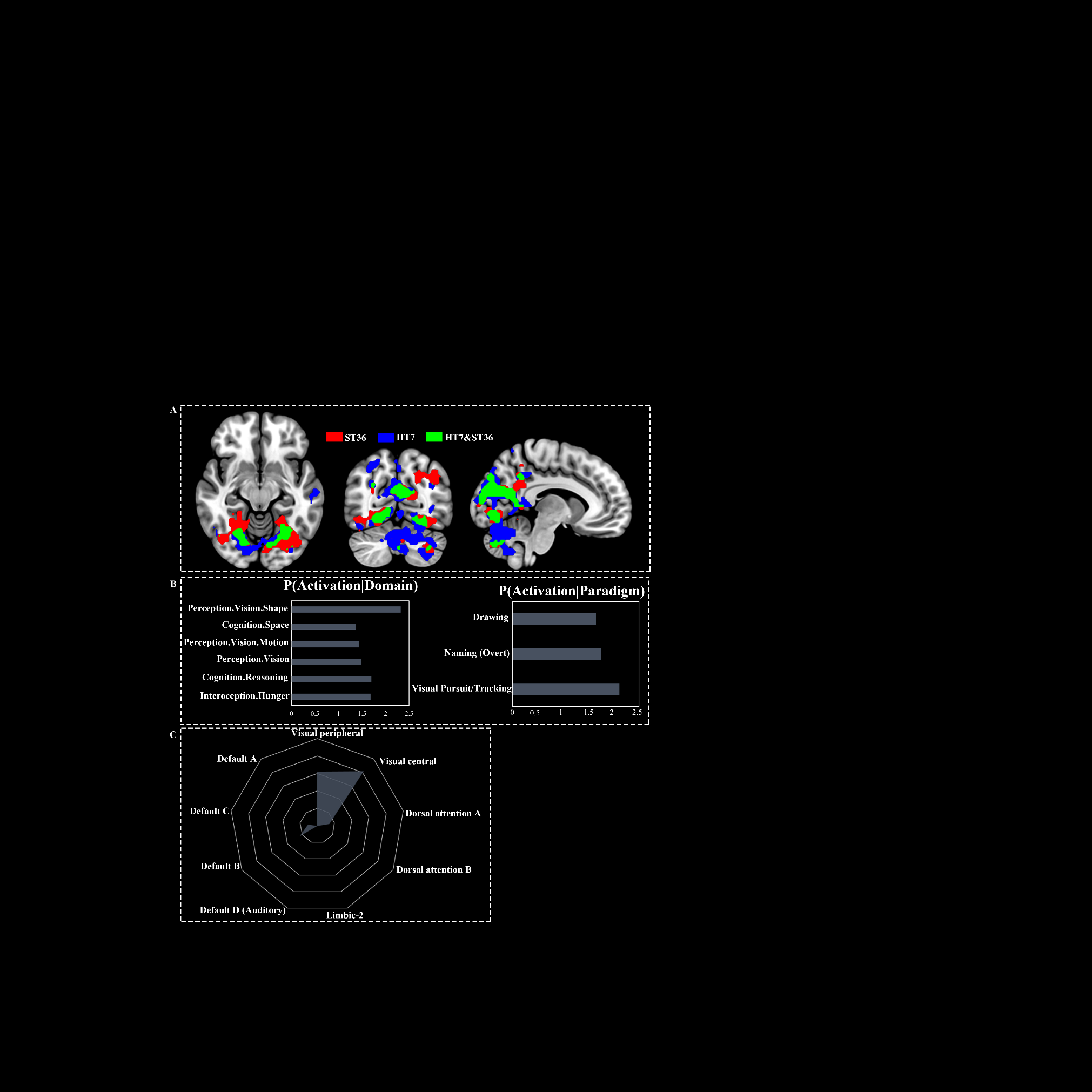


sFigure3 Overlap between HT7 and ST36 activation regions. Panel A illustrates the overlap in activation regions between HT7 and ST36; Panel B highlights the functional characteristics of the commonly activated areas; Panel C displays the network components of these shared activation regions.
